# High-Performance Gel Design for Flexible Pressure-Sensing Films in Taekwondo Applications

**DOI:** 10.3390/gels12030244

**Published:** 2026-03-13

**Authors:** Zhiyong Zhang, Weimin Pan, Qianle Zhang, Yi Men, Niankun Zhang, Tao Liu

**Affiliations:** 1 School of Sports and Health Sciences, Xi’an Physical Education University, Xi’an 710068, China; panweimin@163.com (W.P.); zhangqianle2026@163.com (Q.Z.); 230401005@stu.xaipe.edu.cn (Y.M.); znkwq@163.com (N.Z.); 2 Universities Engineering Research Center of Innovative Technology of Intelligent Sports Equipment in Shaanxi Province, Xi’an 710068, China

**Keywords:** Taekwondo, electronic protective gear, sensing films, ionogel, stress–strain

## Abstract

Exploring effective training methods to reliably trigger scoring in electronic protective gear is a significant challenge faced by coaches and athletes, and it constitutes a critical research direction that urgently demands scientific exploration. To improve the scientific precision of daily Taekwondo training and enhance competitive performance more efficiently and to improve the effectiveness of daily Taekwondo training and enhance competitive performance, a hydrogel-based flexible pressure-sensing film was developed. This film would enable traditional Taekwondo protective gear with electronic sensing capabilities via a simple adhesion method. By attaching a low-cost, high-precision, and appropriately flexible gel-based pressure-sensing film to conventional protective gear through a straightforward adhesion approach, it can attain sensing performance comparable to that of specialized competition-grade electronic protective gear. This innovation will provide technological support for advancing the scientific rigor of Taekwondo training in China. This study focuses on the design and development of high-strength, high-toughness ionic hydrogels, offering technical backing for the creation of flexible pressure-sensing films tailored for Taekwondo applications.

## 1. Introduction

Taekwondo is a combat sport characterized by direct physical contact and high-intensity competition, and it is an official event in the Olympic Games. To ensure fairness, impartiality, and real-time scoring in Taekwondo competitions, the World Taekwondo Federation first decided to introduce electronic protective gear into competitions at the 18th Asian Taekwondo Championships in 2008. Since then, all official Taekwondo competitions have adopted electronic gear to assist in scoring. The validity of strikes is primarily assessed in two approaches. Firstly, points scored by punches to valid target areas are judged and awarded by referees. Secondly, points from kicks to valid target areas are determined by sensors embedded in the protective gear. These sensors quantify the force of impact when the foot protector strikes the opponent’s body or head protector, thereby determining the validity of the strike. This approach acts as the primary scoring mechanism in competitions, rendering electronic scoring the “key player” in determining victory or defeat [1,2,3]. Currently, DaeDo and KP&P are the two officially recognized electronic protective gear brands by the World Taekwondo Federation (WT), and they are alternately utilized across all levels of Taekwondo competitions.

The full set of sensor-integrated electronic protective gear employed in Taekwondo competitions is relatively costly and has a limited service life. Additionally, the supporting software is frequently updated and often fails to meet the requirements of daily Taekwondo training. Studies have indicated that sports teams nationwide procure only a limited number of electronic protective gear sets, with athletes predominantly training with traditional, non-sensor-equipped gear. However, when training with traditional gear, numerous techniques that athletes perceive as score-worthy are often not validated by electronic scoring systems. The primary reason for this is athletes’ inadequate understanding of the operational principles of electronic sensors. This results in a high incidence of ineffective or excessively forceful techniques in competitions and practical training. Ineffective techniques waste athletes’ energy without securing points, whereas excessively forceful actions can disrupt balance, delay posture recovery, and hinder the execution of follow-up techniques all of which diminish scoring efficiency. It is common in competitions to observe cases where an attacking athlete strikes a valid scoring area on the opponent’s gear with a clear, audible impact (a technique that would typically be scored by human judges), yet the electronic system fails to award points. Even athletes who excel in training often a low attain low scores within the allotted competition time. This suggests that numerous techniques practiced in training do not satisfy the criteria for electronic scoring validation, thus failing to effectively improve competitive performance. Consequently, the efforts invested in daily training yield limited outcomes, leading to considerable frustration for both coaches and athletes.

Presently, electronic protective gear is mandatory equipment for Taekwondo World Championships and the Olympic Games. However, national sports teams and Taekwondo associations worldwide maintain a large inventory of traditional gear but possess only a limited number of electronic sets. Consequently, athletes predominantly train with traditional gear, presenting a major challenge for coaches and athletes to develop training methods that can effectively simulate the scoring mechanisms of electronic gear. This issue also represents a critical research direction that urgently requires attention in the field of sports science. Therefore, to enhance the scientific rigor of daily Taekwondo training and improve its effectiveness in boosting competitive performance, there is a need to develop a hydrogel-based flexible pressure-sensing film that can enable traditional Taekwondo gear to acquire electronic sensing capabilities. Using the simplest adhesion methods, this low-cost, high-precision, and suitably flexible hydrogel pressure-sensing film can be attached to traditional gear, endowing it with sensing functions identical to those of specialized electronic competition gear. This advancement would provide technological support for elevating the scientific standards of Taekwondo training.

As a flexible sensing technology based on advanced materials, hydrogel-based flexible pressure-sensing films are highly suitable for flexible electronic device design owing to their superior elasticity, extensibility, and transparency. Hydrogel sensors possess superior extensibility and elasticity, which minimize their interference with Taekwondo protective gear and avoid disrupting athletes during training. Researchers have achieved significant progress in investigating the related properties of hydrogel flexible sensors. For instance, Ruya et al. developed a flexible all-fabric supercapacitive wearable pressure sensor based on an elastic ion-electron interface, which greatly enhanced the sensitivity of ionic pressure sensors and provided technical insights for designing flexible pressure-sensing films [4]. Yan et al. proposed the utilization of quadruple hydrogen-bond-crosslinked supramolecular polymer materials as substrates for stretchable, tear-resistant, and self-healing film electrodes, enabling self-healing functionality in flexible materials and offering technical guidance for hydrogel self-healing design [5]. Wang et al. employed interfacial bonding techniques to combine high-modulus fibers with low-modulus matrices, creating a composite material with high toughness and low hysteresis. This approach achieved the physical properties of high toughness and low hysteresis in elastic materials like hydrogels, providing technical references for enhancing the toughness and accuracy of flexible sensors [6]. Deng et al. studied novel self-deformable soft-hard composite hydrogel films, offering new ideas for designing sensors with special properties [7]. Lee et al. designed a high-performance transparent polymer film [8], while Yan et al. developed a transistor film capable of high-fidelity monitoring and local amplification of potential and electrophysiological signals, providing technical references for designing thin-film transistors in hydrogel films [9]. Wang et al. designed an ionogel with high toughness, shape memory, and self-healing properties, offering technical insights for the design of anti-dynamic fatigue hydrogel devices in this study [10]. Xie et al. developed a mussel-inspired hydrogel with conductivity, long water retention, and self-healing functions [11]. Zhang et al. conducted research on hydrogel toughness, providing theoretical guidance for improving the toughness of hydrogel devices [12]. He et al. designed a high sensitivity and large linearity graphene pressure sensor, which was fabricated into a wearable device capable of monitoring subtle humidity fluctuations in the human body caused by activities such as respiration in a non-contact manner [13]. Gao et al. designed a flexible transparent hydrogel sensor for monitoring finger movements [14]. Chen et al. designed a self-healing hydrogel sensor with multiple shape memory properties for human motion monitoring [15]. Liu et al. developed a super-strong reversible ionic crystal-based adhesive inspired by ice adhesion, proposing a novel design of detachable adhesive suitable for dynamic and complex environments [16]. International researchers have made significant advancements in hydrogel research. Regarding hydrogel flexible devices, they have successfully designed and conducted preliminary experiments for artificial skin, artificial nerves, electroluminescent devices, electro-optical devices, touchscreens, and GEO devices, among others [17,18,19,20,21,22,23,24]. Although sensor devices involved in these studies, such as High-stretchability transparent polymer film [25], ionogels with self-healing properties [26,27], and highly sensitive hydrogel sensors [28,29], provide technical support for the design of flexible pressure-sensing hydrogel films. However, existing studies, particularly those focused on enhancing the mechanical properties of hydrogels, have mostly concentrated on testing tensile mechanical properties. In contrast, the sport of Taekwondo requires hydrogels with good compressive properties. Therefore, current research cannot yet meet the demands of Taekwondo training.

Ionogels possess excellent ionic conductivity [30], thermal stability, electrochemical stability, and non-volatility, making them highly suitable for designing flexible pressure-sensing films for Taekwondo. However, existing ionogels mainly focus on the design of high-performance tensile mechanical properties, while Taekwondo requires superior compressive performance. “Although significant progress has been made in enhancing the tensile properties and self-healing capabilities of ionogels [10,25,26,27], their compressive mechanical performance—a critical requirement for withstanding high-impact forces in sports equipment—has received far less attention. This gap limits their practical application in fields such as Taekwondo, where materials must endure repeated and intense compressive loading without failure.” Consequently, current research cannot yet satisfy the need for high-precision force monitoring in Taekwondo training. Therefore, this paper carries out the design and development of a high-strength, high-compression-toughness ionogel to provide technical support for the development of flexible pressure-sensing films intended for Taekwondo applications. The development of this copolymer ionogel flexible pressure-sensing film, which possesses high compressive resistance, high stability, and high sensitivity, is of great significance for advancing the scientization of Taekwondo training.

## 2. Results and Discussion

### 2.1. Taekwondo Electronic Protective Gear Threshold Patterns

In Olympic Games and National Games, Taekwondo events comprise eight weight categories: women’s 49 kg, women’s 57 kg, women’s 67 kg, women’s +67 kg, men’s 58 kg, men’s 68 kg, men’s 80 kg, and men’s +80 kg. The pressure threshold required for a valid scoring hit on the electronic protectors varies for each weight category.

Experimental tests yielded pressure thresholds for different levels, as shown in Table 1. Since the electronic protective gear relies on piezoelectric film sensors to detect force values, its working principle involves measuring the pressure exerted by athletes’ kicks or strikes on the gear during matches to determine the validity of the action and enable automated scoring. Therefore, the pressure values for each level in the table were expressed in terms of pressure intensity. Table 1 presents only the minimum pressure intensity required for valid scoring. Thus, the performance of the high-performance ionic hydrogel used as the base material for the Taekwondo flexible pressure-sensing film must exceed the tested minimum pressure intensity. This ensures that the flexible pressure-sensing film meets testing requirements while avoiding issues such as fracture or damage during testing, which could affect performance. Consequently, the gel material must possess high fracture strength (above 1.07 MPa). During training, the hydrogel flexible pressure-sensing film will inevitably undergo repeated cycles of stress loading and unloading. Using a hydrogel with high toughness and self-healing properties ensures the stability of the test signals. This requires the hydrogel to exhibit both high compressibility and self-repairing capabilities.

### 2.2. Design of Copolymer Ionogel

**Figure 1:**
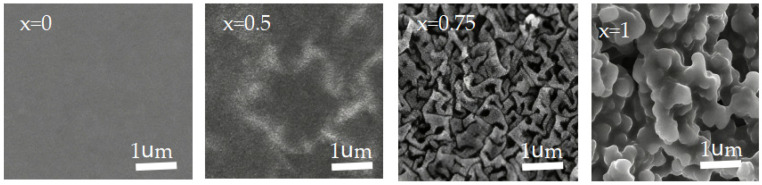
SEM images of various ionogels.

**Figure 2:**
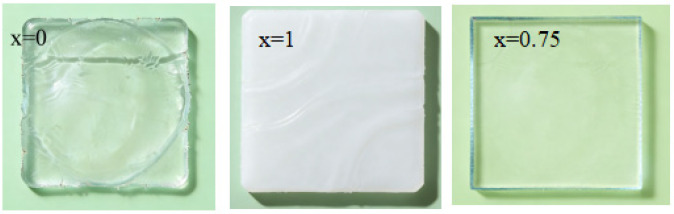
Photographs of various ionogels.

**Figure 3:**
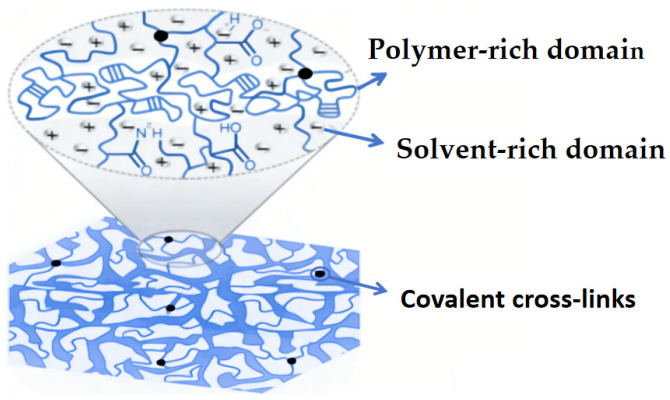
Schematics of copolymer ionogel.

We fabricated a series of copolymer ionogels with the nomenclature P(AAm_x_-co-AA_1−x_), where x represents the molar fraction of AAm in copolymer ionogel. The total monomer concentration (Cm) of AAm and AA was 3 M and the concentration of MBAA (CMBAA) was 0.2 mol%. When x = 0, it was pure PAA; when x = 1, it was pure PAAm. PAA has good compatibility with EMIES (Figure 1 x = 0), and it is transparent (Figure 2 x = 0). PAAm has poor compatibility with EMIES (Figure 1 x = 1),and it is transparent opaque(Figure 2 x = 1). For the copolymer ionogel, a single network containing both polymer-rich and solvent-rich domains can be obtained by randomly copolymerizing AAm and AA monomers in EMIES (Figure 3). According to Figure 2 and Figure 3, it could be seen that, in copolymer ionogel, the polymer-rich phases connect the solvent-rich phases to form a bicontinuous phase network structure.

### 2.3. Mechanical Response of Different Gels

During Taekwondo competitions, electronic protective gear primarily endures striking pressure, requiring hydrogel materials to exhibit both high compressive toughness and effective mechanical responsiveness.

**Figure 4:**
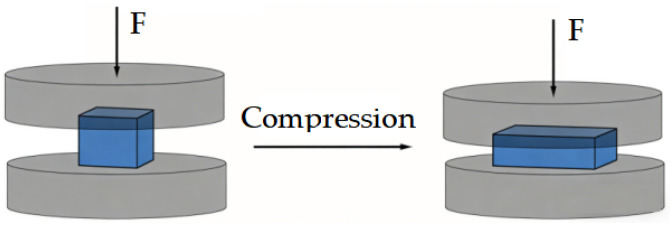
Schematic diagram of the performance testing of various ionogels.

The ionogel was cut into 20 mm × 20 mm specimens and compressed at a rate of 1 mm/min to test its compressive properties. A schematic diagram of the performance testing of various ionogels is shown in Figure 4.

**Figure 5:**
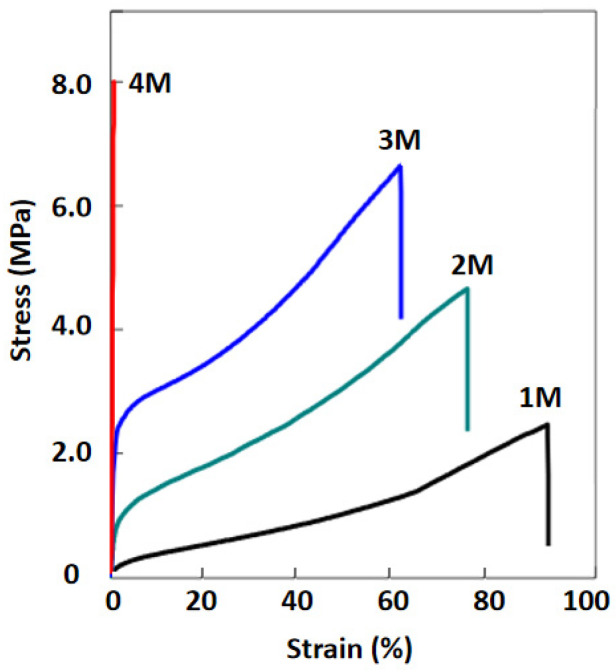
Tensile stress-strain curves of ionogels with four different monomer compositions.

To optimize the mechanical properties, copolymer ionogels with varied Cm were synthesized. As shown in Figure 5, when varying Cm from 1 M to 4 M, the fracture strength of the copolymer ionogels improved from 2.2 to 8.1 MPa. However, the compressive strain at break of the copolymer decreased accordingly, especially at 4 M, where it plummeted to about 2.5%. This property probably results from the high solid content (75.7 wt% at 4 M), which leads to dense phase separation and makes the ionogel stiff and brittle. Among the compositions explored in this study, a Cm value of 3 M (corresponding to a liquid content of approximately 82 wt% in the network) was able to achieve a relatively good compressive fracture strength, attaining an optimized effect.

**Figure 6:**
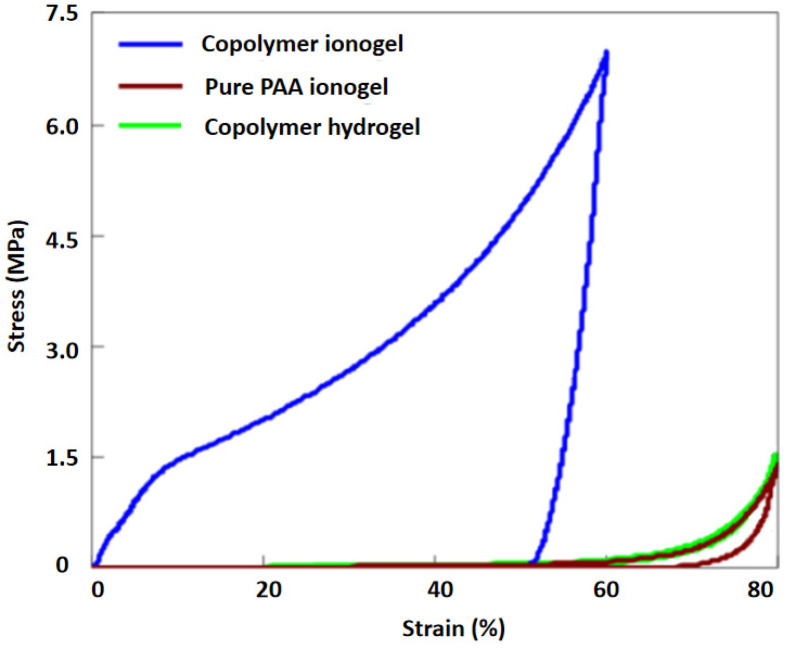
Compression stress–strain diagrams of different gels.

Based on existing research, this study evaluated the performance of four types of gels: copolymer ionogel, pure PAA ionogel, pure PAAm ionogel, and copolymer hydrogel. Testing revealed that the pure PAAm ionogel was excessively fragile, making it unsuitable for testing compressive strength and thus inappropriate as a material for Taekwondo flexible pressure-sensing films. Therefore, Figure 6 presents only the compressive performance of the remaining three gels: copolymer ionogel, pure PAA ionogel, and copolymer hydrogel.

As shown in Figure 6, the copolymer ionogel was able to withstand nearly 60% compressive strain and reaches the maximum range of the testing instrument without failure, demonstrating extremely high compressive strength and deformation resistance. This is primarily attributed to the abundant polymer-rich domains within its structure, which provide robust rigid support, while the solvent-rich phases in the ionogel effectively disperse the applied load. This synergistic effect of rigid support and load dispersion endows the material with excellent compressive and deformation resistance. In contrast, the pure PAA ionogel exhibits a compressive strength of 1.5 MPa at 80% strain, showing relatively weaker compressive performance. This was due to the lack of high-strength polymer-rich domains in its network structure, making it difficult to effectively resist compressive loads. The copolymer hydrogel, with a compressive strength of 1.7 MPa at 80% strain, demonstrates better compressive performance than the pure PAA ionogel but remains inferior to the copolymer ionogel. Based on existing studies [10], this was mainly because the solvent (water) in the copolymer hydrogel was easily extruded under compression, leading to rapid collapse of the network structure and limiting its compressive strength.

From the results of the Effective striking thresholds for each level in Taekwondo in Table 1, it can be seen that the gel material was required to possess a high fracture strength exceeding 1.07 MPa, while also exhibiting high compression resistance. The results indicate that both the pure PAA ionogel and the copolymer hydrogel require significant deformation to meet the high fracture strength condition required for Taekwondo electronic protective gear testing. However, during Taekwondo competitions, there were instances where the impact force exceeds the fracture limit of these two materials. Therefore, the analysis reveals that the copolymer ionogel has a greater advantage in the design of flexible pressure-sensing films for Taekwondo, exhibiting stronger compressive capacity and being able to meet the kicking requirements of all Taekwondo weight classes. Consequently, using the copolymer ionogel as the main material for designing Taekwondo flexible pressure-sensing films is more advantageous.

### 2.4. Mechanical Response Patterns of Ionogels with Different Monomer Ratios

In addition to meeting the requirement of high compressive toughness, gels must also possess fatigue resistance. Based on the theory of dissipation-induced toughening, [32] this study adopts a strategy of high copolymerization and low solubility monomers. By forming a dual-continuous synergistic structure within a single network—comprising low-solvation (hydrogen bond-enriched) domains and high-solvation (elastic gel-like) domains—the synthesized copolymer ionogel was designed to exhibit excellent fatigue resistance (under repeated loading) and toughness, thereby effectively meeting the testing demands of Taekwondo training.

Existing research indicates that variations in concentration and chemical composition in copolymer ionogels can lead to different mechanical effects. This study investigates the mechanical response patterns of ionogels by adjusting the ratios of PAAm and HEMA at different concentrations. The proportions of different components in the copolymer ionogels with varying ratios were detailed in Table 2.

**Figure 7:**
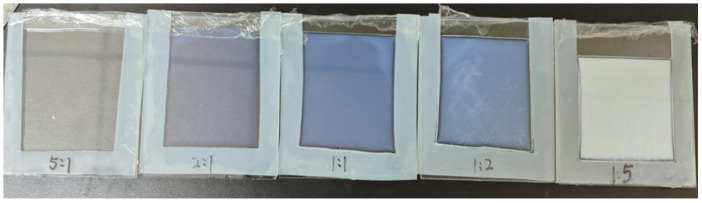
Photographs of copolymer ionogels with different component ratios.

As shown in Table 2, five different ratios of polyacrylamide (PAAm) to hydroxyethyl methacrylate (HEMA) were set: 5:1, 2:1, 1:1, 1:2, and 1:5. The images of the copolymer ionogels with different component ratios were shown in Figure 7. This study investigates the mechanical response sensitivity patterns of gel materials under these five different proportion conditions. As shown in Figure 3, the stress–strain patterns of gels with different monomer ratios under repeated cyclic loading were illustrated.

**Figure 8:**
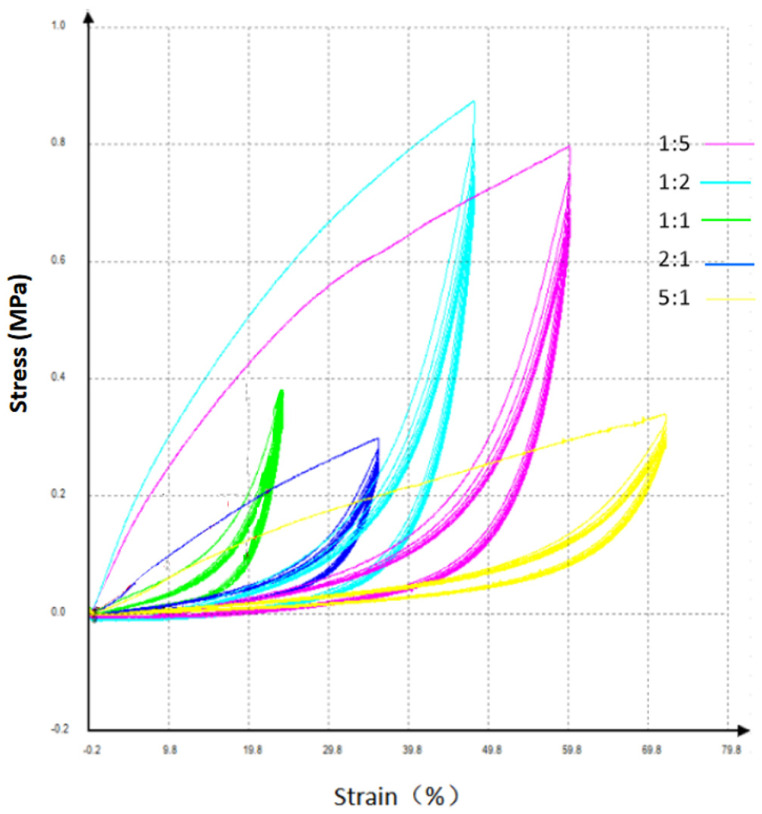
Stress–strain behavior of copolymer ionogels with different monomer ratios under repeated cyclic loading.

As shown in Figure 8, cyclic loading experiments reveal that different monomer ratios in copolymer ionogels lead to varying mechanical response sensitivities. After 100 cycles of repeated loading tests, the stress–strain behavior of copolymer ionogels with each monomer ratio remained largely consistent during the cyclic loading process. The only slight variation observed was the hysteresis during the unloading phase, primarily attributed to the viscoelastic nature of gels, which inherently exhibit such hysteresis effects during unloading. This minor difference does not affect the testing requirements. Overall, the copolymer ionogels demonstrated stable performance and strong resistance to repeated loading, indicating excellent anti-fatigue properties and sufficient stability to meet testing needs.

As shown in Figure 8, among the five monomer ratio schemes adopted in the experiment, the AAm-to-HEMA ratio of 1:2 demonstrates a more pronounced rate of change in stress–strain, which was beneficial for enhancing the performance of the Taekwondo flexible pressure-sensing film. When the AAm-to-HEMA ratio exceeds 1:1, the rate of change in stress–strain gradually decreases as the ratio coefficient further increases. Therefore, in the design process of the Taekwondo flexible pressure-sensing film, emphasis should be placed on adopting a preparation scheme with a lower AAm-to-HEMA ratio to improve the testing accuracy of the sensing film.

Analysis of the threshold patterns for Taekwondo electronic protective gear reveals that the pressure thresholds required for valid scoring across all competition categories in the Olympic Games and the National Games are primarily concentrated between 0.6 MPa and 1.07 MPa. Striking forces exceeding 1.07 MPa can be judged as valid scores in any competition category, while forces below 0.6 MPa cannot achieve valid scores in any category. Therefore, in the design and research of high-performance gels suitable for Taekwondo flexible pressure-sensing films, emphasis should be placed on the mechanical response and sensitivity of gels within the pressure range of 0.6 MPa to 1.1 MPa. The stress–strain characteristics observed at an AAm-to-HEMA ratio of 1:2 align more closely with the demands of Taekwondo competitions.

**Figure 9:**
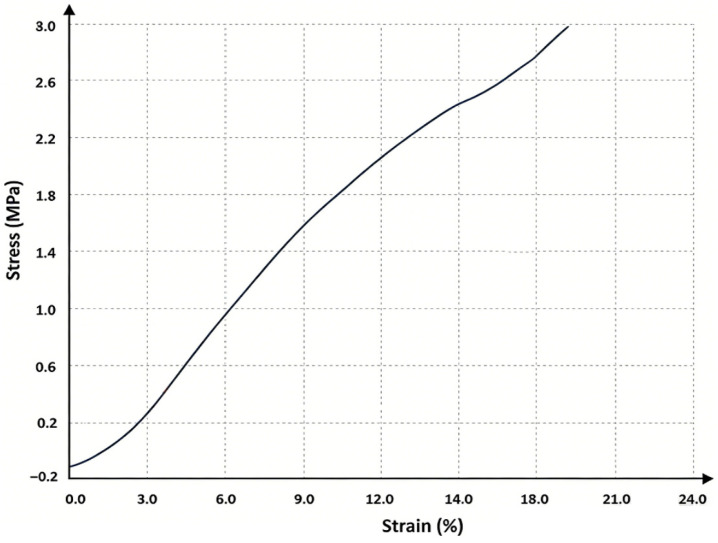
Stress–strain plot of 1:2 AAm/HEMA.

As shown in Figure 9, the copolymer ionogel with an AAm-to-HEMA ratio of 1:2 exhibits excellent linear correlation between stress and strain under compressive loading. This correlation was particularly pronounced within the stress range of 0.6 MPa to 1.1 MPa, making it highly suitable for the design of Taekwondo pressure-sensing films. Additionally, the copolymer ionogel demonstrates extremely high compressive strength and deformation resistance. Even when subjected to intense kicking forces, the sensing film remains undamaged, making it ideal for force testing and monitoring during striking actions.

## 3. Conclusions

In this study, a high-performance copolymer ionogel was designed and developed as a flexible pressure-sensing film for Taekwondo applications, addressing the specific need for high compressive toughness and mechanical stability in electronic protective gear. The key findings and contributions are summarized as follows:

Material Design and Structural Insight: A series of copolymer ionogels P(AAm_x_-co-AA_1−x_) were successfully synthesized via a one-step random copolymerization in EMIES. By leveraging the poor compatibility of PAAm with the ionic liquid, a bicontinuous network structure comprising polymer-rich and solvent-rich phases was formed. This unique architecture provides synergistic rigid support and load dispersion, which is crucial for achieving high compressive performance.

Superior Mechanical Performance: The optimized copolymer ionogel (with a Cm of 3 M and an AAm:HEMA ratio of 1:2) exhibits exceptional compressive properties. It can withstand nearly 60% strain without failure and demonstrates a record-high compressive strength within the context of Taekwondo requirements. Compared to pure PAA ionogels and copolymer hydrogels, the copolymer ionogel shows significantly higher fracture strength and deformation resistance, making it the most suitable candidate for withstanding the high-impact forces in Taekwondo competitions.

Application-Oriented Validation: Threshold analysis of official Taekwondo electronic protective gear (0.6–1.07 MPa) confirms that the copolymer ionogel meets the force requirements across all weight categories. Its stable mechanical response and excellent linear stress–strain correlation within the critical 0.6–1.1 MPa range demonstrate its high sensitivity and reliability for real-time scoring and monitoring in training.

Fatigue Resistance and Stability: Cyclic loading tests reveal that the copolymer ionogel possesses excellent anti-fatigue properties, maintaining consistent mechanical response after repeated compression. This ensures the long-term durability and accuracy of the sensing film during intensive training sessions.

In conclusion, the developed copolymer ionogel represents a promising base material for next-generation flexible pressure-sensing films in Taekwondo. Its high compressive toughness, sensitivity, and stability effectively bridge the gap between daily training with traditional gear and the precision required for electronic competition scoring. While this study focuses on meeting practical application demands, future work will aim to achieve greater conceptual and materials innovation, including a deeper investigation into energy dissipation mechanisms and the exploration of new polymer architectures to further enhance performance.

## 4. Materials and Methods

### 4.1. Selection of High-Toughness Gels

Based on existing research [10], this study primarily selected four types of gels for comparative performance analysis: copolymer hydrogel, pure polyacrylic acid (PAA), pure polyacrylamide (PAAm), and copolymer ionogels (PAAm-co-PAA). Copolymer hydrogel, pure PAA, pure PAAm, and copolymer ionogels (PAAm-co-PAA) were all prepared through covalent cross-linking using N,N′-methylenebisacrylamide (MBAA). Among these, the copolymer ionogels were synthesized by randomly copolymerizing AAm and AA monomers in EMIES. The polymer-rich phase forms a bicontinuous network structure by penetrating the solvent-rich phase, thereby effectively synergizing to enhance the mechanical properties of the material.

### 4.2. Performance Testing of Taekwondo Electronic Protective Gear

To design high-performance gels suitable for Taekwondo flexible pressure-sensing films, it was essential first to test the sensor distribution, force-response patterns, and threshold ranges across different levels of specialized Taekwondo electronic protective gear (such as DaeDo and KP&P) (Daedo International, Barcelona, Spain and KPNP, Seoul, Republic of Korea). This analysis aims to determine the fundamental performance parameters of ionic hydrogels, ensuring that the designed hydrogel flexible pressure-sensing film better meets practical requirements. This study employs an integrated testing system consisting of a three-dimensional force platform (Kistler) (Kistler Group, Winterthur, Switzerland) and a high-speed camera system (Sony Corporation, Tokyo, Japan), synchronized with the electronic scoring system. In the experiments, shot puts of varying weights and volumes were used as force sources by dropping them from different heights in free fall. During testing, Taekwondo protective gear was placed on the three-dimensional force platform (Kistler), with a foot guard positioned on top. A spatial coordinate system was established, and the electronic protective gear was divided into over a thousand small sections for individual testing. Small balls were dropped onto the electronic gear in free fall, with repeated tests conducted at varying heights. The entire process was recorded using a high-speed camera.

### 4.3. Testing of Gel Properties

To investigate high-performance gels suitable for pressure-sensing films that meet the training demands of Taekwondo, this study employed an electronic universal testing machine (Wance TSE104C) (Wance, Shenzhen, China) to conduct various mechanical property tests on the gels. The film samples were cut into 20 mm × 20 mm specimens and compressed at a rate of 1 mm/min. Given that protective gear primarily experiences compressive loads from kicks and strikes during Taekwondo competitions, this research was designed to test the compressive strain, pressure resistance, and mechanical response patterns of different gels.

## Figures and Tables

**Table 1:** Effective striking thresholds for each level in Taekwondo.

Competition Weight Categories *	Electronic Protector Display Threshold	Pressure (MPa)	Striking Speed (m/s)
women’s 49 kg	16	0.60	2.47
women’s 57 kg	18	0.71	2.84
women’s 67 kg	20	0.87	3.34
women’s +67 kg	21	0.88	3.54
men’s 58 kg	20	0.87	3.37
men’s 68 kg	21	0.88	3.37
men’s 80 kg	23	1.00	3.94
men’s +80 kg	25	1.07	4.20

* The competition weight categories data was sourced from the official Olympic Games website [31]. The other data were obtained through experimental testing. For the testing methods, refer to [Section 4.2].

**Table 2:** Preparation scheme for copolymer ionogels with different component ratios.

Monomer	Molecular Weight	AAm:HEMA (5:1)	AAm:HEMA (2:1)	AAm:HEMA (1:1)	AAm:HEMA (1:2)	AAm:HEMA (1:5)
AAm	71.079	1.7769	1.4215	1.0662	0.7108	0.3554
HEMA	130.14	0.6507	1.3014	1.9521	2.6028	3.2535
MBAA (0.1 mol%)	154.17	0.0046	0.0046	0.0046	0.0046	0.0046
OA (0.05 mol%)	146.1	0.0021	0.0021	0.0021	0.0021	0.0021
H_2_O	18.01528	9.3493	8.6986	8.0479	7.3972	6.7465

## Data Availability

The original contributions presented in the study are included in the article; further inquiries can be directed to the corresponding author.
